# Mast cell amines and inosineinduced vasoconstriction in the rat hind limb

**DOI:** 10.1080/09629359791848

**Published:** 1997-04

**Authors:** A. M. Northover, B. J. Northover

**Affiliations:** 1 Department of Pharmaceutical Sciences School of Applied Sciences De Montfort University Leicester LE1 9BH UK

## Abstract

Under certain circumstances injected inosine causes a net vasoconstrictive effect on the arterioles, which has been attributed to 5-hydroxytryptamine (5HT) released in response to adenosine type 3 (A_3_) receptor stimulation of mast cells residing in the adventitia. We have sought further evidence for this hypothesis using blood vessels of the rat hind limb perfused in vitro at constant rate with a gelatin-containing physiological salt solution. Injection of inosine (2.7 mg) caused a rise in perfusion pressure, which was only slightly increased by inclusion of N-nitro-L-arginine methyl ester (100 μM) in the perfusate. Inclusion in the perfusate of cyproheptadine (1 μM), compound 48 80 (1 μg ml), 8-phenyltheophylline (1 μM) or 8-cyclopentyl-1,3 dipropylxanthine (0.1 μM) greatly reduced the pressor response to inosine. The pressor effect of injected 5HT (400 μg) was abolished by pre-treatment with cyproheptadine, but not by pre-treatment with compound 48 80. These results suggest that the net pressor response to injected inosine was mainly the result of an A_1_ receptor-mediated release of 5HT, most probably from mast cells. No evidence was found for an involvement of A_3_ receptor stimulation.


Mediators of Inflammation, 6, 141–145 (1997)

### Mast cell amines and inosine-induced vasoconstriction in the rat hind limb

A. M. Northover[CA] and B. J. Northover

Department of Pharmaceutical Sciences, School of Applied Sciences, De Montfort University, Leicester LE1 9BH, UK

[CA]Corresponding Author
Fax: (+44) (0) 116 2577135

**UNDER certain circumstances injected inosine causes a net vasoconstrictive effect on the arterioles, which has been attributed to 5-hydroxytryptamine (5HT) released in response to adenosine type 3 ($A_3$) receptor stimulation of mast cells residing in the adventitia. We have sought further evidence for this hypothesis using blood vessels of the rat hind limb perfused *in vitro* at constant rate with a gelatin-containing physiological salt solution. Injection of inosine (2.7 mg) caused a rise in perfusion pressure, which was only slightly increased by inclusion of N-nitro-L-arginine methyl ester (100 μM) in the perfusate. Inclusion in the perfusate of cyproheptadine (1 μM), compound 48/80 (1 μg/ml), 8-phenyltheophylline (1 μM) or 8-cyclopentyl-1,3 dipropylxanthine (0.1 μM) greatly reduced the pressor response to inosine. The pressor effect of injected 5HT (400 μg) was abolished by pre-treatment with cyproheptadine, but not by pre-treatment with compound 48/80. These results suggest that the net pressor response to injected inosine was mainly the result of an $A_1$ receptor-mediated release of 5HT, most probably from mast cells. No evidence was found for an involvement of $A_3$ receptor stimulation.**

**Key words:** Adenosine receptors, Compound 48/80, 8-Cyclopentyl-1,3 dipropylxanthine, Cyproheptadine, 5-Hydroxytryptamine, Inosine, Mast cell amines, 8-Phenyltheophylline

### Introduction

Despite the fact that adenosine generally acts as a vasodilator in mammalian vascular beds,[1] both adenosine and its main metabolite inosine have been shown to cause vasoconstriction in the rat perfused hind limb[2] and in hamster cheek pouch arterioles.[3,4] Adenosine can also contract strips isolated from rat tail arteries.[5] Such responses have been shown to involve the release of mast cell amines,[2–5] and may result from stimulation of the adenosine type 3 ($A_3$) receptors,[6] that have been demonstrated to occur in mast cells.[7] In the present work we have attempted to discover the type of adenosine receptor which is responsible for inosine-induced vasoconstriction in the rat perfused hind limb. A gelatin-containing physiological salt solution (GPSS) was used as perfusate, rather than blood, in order to eliminate the possible effects of 5-hydroxytryptamine (5HT) which may be released from rat platelets.[8] Inosine was used in preference to adenosine since previous workers have shown that arterioles constrict in response to inonsine over a wide range of concentrations, whereas adenosine constricts at low concentrations but at higher concentrations may dilate blood vessels.[3] The types of receptor involved have been explored with the aid of various adenosine and 5HT receptor agonists and antagonists.

### Methods

Female rats weighing approximately 250 g were killed by inhalation of chloroform vapour. The abdomen and thorax were opened using a ventero-lateral incision after which the vasculature was heparinized via an intra-cardiac injection. Subsequently, the left iliac artery, both renal arteries and the aorta proximal to the renal arteries were all ligated. A stainless steel cannula (1.07 mm OD), which offered no measurable resistance to flow at 10 ml/min, was then inserted into the right iliac artery via an incision in the aorta distal to the renal arteries, and was tied in place. Perfusate was then pumped into the right iliac artery at a rate of

© 1997 Rapid Science Publishers

10 ml/min, and allowed to drain away through a hole cut in the inferior vena cava. Perfusion pressures were recorded from a Condon manometer connected to the perfusion line and via a strain gauge pressure transducer connected to a Multitrace 2 recorder (Lectromed, UK). A paper speed of 25 mm/min was used for the 1 min period immediately following bolus injections of various adenosine receptor agonists or of 5HT. These were delivered into the stream of perfusate using a back-to-back syringe system to minimize injection artefacts. Compounds to be tested as possible antagonists were added to the GPSS at the start of the experiment. All preparations were perfused for an initial stabilizing period of 10 min. Then, after recording baseline perfusion pressure for 3 min, 2.7 mg inosine in 0.2 ml normal saline (NS, 0.9% NaCl) was injected. Perfusion pressures thereafter were recorded continuously for 5 min. After perfusing for a further 5 min, to allow time for the effects of inosine to cease, the baseline pressure was again recorded and a control injection of 0.2 ml NS was given. Finally, this procedure was repeated with a bolus injection of 5HT (400 μg in 0.2 ml NS). Pilot experiments indicated that these doses of inosine and 5HT were necessary to produce suitable pressor responses. Preliminary experiments showed, as have those of previous workers,[2] that the pressor response to inosine vanishes after approximately three injections have been made into the same preparation. Therefore, only one inosine, one NS and one 5HT response (where relevant) were recorded from each animal.

Experiments were also performed using the rat perfused tail vascular bed. For this, male rats weighing about 350 g were killed and heparinized as above. Both renal arteries and both iliac arteries were ligated in this case. After ligating the aorta proximally, the steel cannula was inserted into the tail artery via the distal aorta. Experiments then followed the protocol described above.

GPSS had the following composition (mM): NaCl, 138; KCl, 5; $NaHCO_3$, 10.1; $MgCl_2$, 1.06; $NaH_2PO_4$, 0.416; $CaCl_2$, 2; glucose, 10; plus 2% gelatin, giving pH 7.4. Perfusates were gassed throughout with 95% $O_2$ and 5% $CO_2$ and were delivered at 37°C.

Compounds added to the GPSS were N-nitro-L-arginine methyl ester (L-NAME), a nitric oxide (NO) synthase inhibitor;[9] cyproheptadine, a mixed histamine and 5HT receptor blocker;[10] compound 48/80, which degranulates mast cells;[11] 8-cyclopentyl-1,3 dipropylxanthine (DPCPX), a relatively specific $A_1$ receptor antagonist;[12] or 8-phenyltheophylline (8PT), a mixed $A_1/A_2$ receptor antagonist.[13] In addition to inosine two other putative adenosine receptor agonists were tested in the hind limb. There were $N^6$-cyclopentyladenosine (CPA), which acts primarily via $A_1$ receptors,[14] and iodobenzyl-5-N-methyl carboxamidoadenosine (IB-MECA), which shows a much greater affinity for $A_3$ than for either $A_1$ or $A_2$ receptors.[15] These two compounds were administered in dimethyl sulphoxide (DMSO) diluted 1 + 19 with NS. Control injections in such preparations, therefore, were with DMSO + NS (1 + 19) rather than with NS alone.

All pressure recordings were made at the same amplification and paper speed, so they could be compared directly with each other. In order to compensate for any residual injection artefact, reponses to each agonist were expressed (in arbitrary units) as the difference between the trace areas (Fig. 1) displayed above and below the pre-injection baseline, and after subtracting the response area for NS alone (or DMSO + NS) shown in the same preparation. Values for the maximum amplitude (A) reached were determined as shown in Fig. 1.

### Chemicals

Inosine, 5HT creatinine sulphate, DPCPX, compound 40/80, 8PT, and CPA were obtained from Sigma Chemical Co. Ltd (Poole, UK); cyproheptadine HCl from Merck Sharp & Dohme Ltd

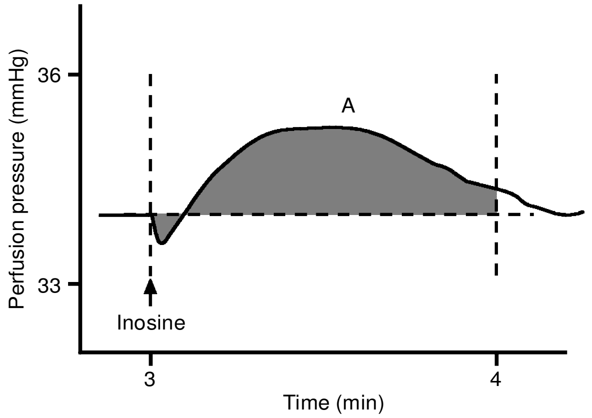

FIG. 1. Diagrammatic representation of a typical pressor response to a bolus injection of inosine (2.7 mg) in the rat hind limb perfused with GPSS + L-NAME (100 μg). Recordings were made on 1 mm squared paper. The pressor response to inosine in NS was calculatd as the difference between the number of squares in the shaded area above the baseline minus the number of squares in the shaded area below the baseline. The equivalent numbers of squares (above and below the baseline) after an injection of NS (0.2 ml) was calculated from the same preparation. The response to NS alone was then subtracted from the response to inosine in NS. Final results were expressed in arbitrary units (Fig. 2 and Table 1). The maximum amplitude of the response (in mm) was determined at A.

(Hoddesdon, UK); and IB-MECA from RBI (Natick, MA, USA).

Inosine and 5HT were dissolved in NS. L-NAME, compound 48/80 and cyproheptadine were added to GPSS as aqueous solutions. DPCPX, 8PT, CPA and IB-MECA were each initially dissolved in DMSO. The final concentration of DMSO in a perfusate was always < 0.05%.

### Statistics

Significant differences ($p < 0.05$) were determined using Bonferroni's test for comparing several treatment groups with one control group.

### Results

### Responses to inosine and 5HT

In experiments using rat hind limb vessels perfused *in vitro* at constant rate with GPSS, a bolus of inosine (2.7 mg) caused a pressor response that began to wane after the first minute. There was no evidence that inosine produced any vasodilatation. A transient fall in perfusion pressure, however, occurred after each injection of NS. Hence this residual injection artefact needed to be compensated for in the responses to inosine and 5HT. Consequently the magnitude of the NS response was routinely deducted from that recorded after giving inosine or 5HT in the same animal. The pressor effect of 5HT (400 μg), unlike that due to inosine, continued to increase slowly for about 5 min. Because of these differing time courses, we decided to present the results obtained using area units. However, the maximum pressor amplitudes attained during the first minute after injection of either inosine or 5HT were closely related to the corrected areas of the pressor responses, but are not separately presented. The pressor effects of inosine and 5HT measured over the first minute post-injection were 54.1 ± 15.3 and 141.8 ± 32.9 area units respectively. Similar results were obtained with the rat tail vessels perfused with GPSS, where the pressor responses to inosine and to 5HT were 17.0 ± 13.9 and 231.6 ± 45.8 area units respectively. Mean starting perfusion pressures with GPSS flowing at 10 ml/min in the hind limb and tail vascular beds were approximately 34 and 70 mmHg respectively. When L-NAME (100 μM) was present in the GPSS perfusing the hind limb, the pressor responses to inosine and to 5HT were increased slightly to values of 61.3 ± 9.6 and 192.0 ± 30.7 area units respectively. This suggests that when NO-synthase was still active a slight dilatory effect from endogenous NO,[16] may have blunted the net vasoconstriction that was produced by both inosine and 5HT. In all subsequent experiments L-NAME was added to the GPSS.

### Effects of cyproheptadine and compound 48/80

Inclusion of cyproheptadine (1 μM) or compound 48/80 (1 μg/ml) in an L-NAME-containing GPSS significantly reduced the pressor effects of both injected inosine (Fig. 2) and 5HT (Table 1), suggesting that the pressor effects of inosine were attributable to 5HT released from a source within or adjacent to the vasculature.

### Effects of specific adenosine receptor antagonists and agonists

When 8PT (1 μM) or DPCPX (1 μM) was included in an L-NAME-containing GPSS the response to inosine in the hind limb vessels was significantly reduced (Fig. 2). The specific $A_1$

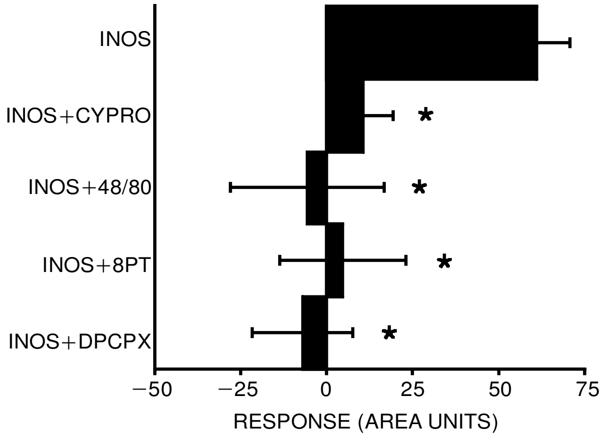

FIG. 2. The vasopressor effect of inosine (2.7 mg) in the rat hind limb, and its modification by pre-treatment with cyproheptadine (cypro, 1 μM), compound 48/80 (1 μg/ml), 8PT (1 μM) or DPCPX (0.1 μM). * $p < 0.05$, Bonferroni's test, compared with the value for inosine alone. n = 7–14.

**Table 1**. Modification of the vasoconstrictive effects of a bolus injection of 5HT (400 μg) on rat hind limb vessels by cyproheptadine (cypro, 1 μM) and compound 48/80 (1 μg/ml)

| Treatment | *n* | *Response (in area units)*[a,b] |
|---|---|---|
| 5HT | 13 | 206.5 ± 29.2 |
| 5HT + cypro | 7 | −9.7 ± 56.3[c] |
| 5HT + 48/80 | 8 | 44.8 ± 30.2[c] |

[a] Results are mean ± SEM.
[b] For details see Methods.
[c] Significant difference compared with 5HT alone ( $p < 0.05$, Bonferroni's test).

receptor agonist CPA (500 μg) caused virtually no change in perfusion pressure ($-0.5 \pm 26.0$ area units, $n = 6$). This was converted to a small depressor effects ($-9.0 \pm 14.3$ area units, $n = 3$) after pre-treatment with compound 48/80 (1 μg/ml), but the difference from CPA responses without compound 48/80 pre-treatment was not statistically significant. The highly potent and specific $A_3$ agonist IB-MECA (5 μg), exerted a statistically insignificant haemodynamic effect using this protocol ($-16.7 \pm 41.8$ area units, $n = 3$).

### Discussion

Most adenosine receptor agonists can relax most types of vascular smooth muscle.[1] This effect is exerted, at least partly, via direct stimulation of both the muscular $A_1$ receptors, which can open both $K_{ATP}$ channels[17,18] and $K_{Ach}$ channels,[18] and via the muscular $A_2$ receptors, which activate adenylyl cyclase.[19] Vasodilatation occurring during periods of ischaemia is associated with, and partly due to, the increased concentrations of adenosine and its metabolite inosine[20] which occur in the vicinity of blood vessels under these circumstances.[21–24] Exogenous inosine can also dilate the coronary blood vessels.[25] The vasoconstrictor responses to inosine which we report here, therefore, although confirming previous work,[2–4] are rather atypical, but not peculiar to the rat, having been observed also in the hamster.[3,4]

The amount of inosine that was required to cause vasoconstriction in the present experiments (2.7 mg) was quite high, but not surprisingly so, since $K_i$ values for inosine at $A_1$, $A_2$ and $A_3$ receptors are reported to be in the 20–50 μM range.[15] Furthermore, about 1 mg of adenosine is required to constrict blood vessels in the rat hind limb during perfusion with a physiological salt solution, whereas 30 μg is sufficient to cause vasoconstriction during perfusion with blood.[26] These same workers have demonstrated the presence of 5HT in venous effluent collected during treatment with adenosine, but they did not speculate upon the site of origin of the 5HT. Perivascular mast cells are one obvious possibility, but coronary artery endothelial cells also contain, and may actually secrete, 5HT.[27] Circulating platelets provide another source of 5HT when blood is used as the perfusate.[8]

Adenosine itself has been shown to stimulate granule/amine release from various types of mast cells.[28–32] Inosine has a similar effect, but only at higher concentrations.[30,32] Moreover, mast cells are normally present in the adventitial layer of various mammalian arterioles.[3,33,34] Since rat mast cell granules contain 5HT[35] adenosine receptor agonists that are delivered via the blood vessel lumen should be able to release 5HT from mast cells in the wall, provided that they can readily penetrate the intimal and medial layers of the wall, or reach the adventitia via the capillaries. Since 5HT is predominantly a vasoconstrictor,[36] it is entirely possible that vasoconstrictor responses to adenosine receptor agonists that are seen in blood vessels of the rat hind limb, are due to released 5HT. Indeed, in the present experiments inosine lost its vasopressor effect after pre-treatment with cyproheptadine or with compound 48/80. Our evidence, therefore, supports a mast cell origin for the 5HT.

If inosine releases 5HT from adventitial mast cells, as the foregoing results would suggest, then it remains to decide which class of adenosine receptor was responsible. The fact that pre-treatment with either 8PT (a mixed $A_1/A_2$ antagonist),[13] or with DPCPX (a selective $A_1$ receptor antagonist),[12] was able to prevent the pressor response to inosine suggests that it was $A_1$ receptor activation which caused the release of 5HT here. Previous work on rat omental mast cells has indicated that degranulation occurs in response to selective $A_1$ receptor agonists,[32] including CPA. However, in the present experiments CPA produced no net effect on the perfusion pressure. This may have been because of a slower penetration of CPA than of inosine through the intimal and media layers of blood vessel walls on its way to the adventitia. In other words, CPA may not have stimulated adventitial mast cells powerfully enough to overcome the direct vasodilatory action which is likely to have been produced by CPA via $A_1/A_2$ receptors on the muscle cells (or the endothelial cells) of the wall. Of relevance in this connection is that on rat isolated aortae CPA was found to exert a net relaxant effect which was attributable to activation of endothelial $A_{2b}$ receptors.[37]

IB-MECA is claimed to be a selective $A_3$ receptor agonist, the $K_i$ values at $A_1$, $A_2$ and $A_3$ receptors being $54 \pm 5$ nM, $56 \pm 8$ nM and $1.1 \pm 0.3$ nM respectively.[15] In contrast, at $A_3$ receptors inosine shows a $K_i$ value approximately 50 000-fold higher than IB-MECA.[15] On this basis, therefore, if inosine had caused its effects in the present experiments via $A_3$ receptors, one would have expected IB-MECA to have mimicked inosine, but at a 50 000-fold lower concentration. In fact, IB-MECA failed to mimic the effect of inosine at a concentration of 1000-fold less than that of the effective dose of inosine. It seems unlikely, therefore, that $A_3$ receptors are involved. Similarly, IB-MECA had no degranulating effect on omental mast cells *in vitro*.[32] Such a lack of response may merely reflect poor tissue penetration. Nevertheless, so far we have failed to obtain any evidence that $A_3$ receptor stimulation was responsible for the release of mast cell amines in our experiments. This contrasts with the findings of some previous workers[6,34] under different experimental conditions. These discrepancies are so far unexplained, but it may be relevant that the IB-MECA-induced hypotension which occurs in cats was attributable to the activation of $A_1$ and $A_2$ receptors rather than of $A_3$ receptors.[38] Hopefully, with the advent of more specific adenosine receptor agonists these divergent observations will be explained.

### References

1. Haddy FJ, Scott JB. Metabolically linked vasoactive chemicals in local regulation of blood flow. *Physiol Rev* 1968; **48**: 688–707.
2. Sakai K, Akima M. Vasoconstriction after adenosine and inosine in the rat isolated hindlimb abolished by blockade of tryptaminergic mechanisms. *Naunyn-Schmiedeberg's Arch Pharmacol* 1978; **302**: 55–59.
3. Doyle MP, Linden J, Duling BR. Nucleoside-induced arteriolar constriction: a mast cell-dependent response. *Am J Physiol* 1994; **266**: H2042–H2050.
4. Shepherd RK, Duling BR. Inosine-induced vasoconstriction is mediated by histamine and thromboxane derived from mast cells. *Am J Physiol* 1996; **270**: H560–H566.
5. Brown CM, Collis MG. Adenosine contracts the isolated rat tail artery by releasing endogenous 5-hydroxytryptamine. *Eur J Pharmacol* 1981; **76**: 275–277.
6. Shepherd RK, Linden J, Duling BR. Adenosine-induced vasoconstriction *in vivo*. Role of the mast cell and $A_3$ adenosine receptor. *Circ Res* 1996; **78**: 627–634.
7. Ramkumar V, Stiles GL, Beaven MA, Ali H. The $A_3$ adenosine receptor is the unique adenosine receptor which facilitates release of allergic mediators in mast cells. *J Biol Chem* 1993; **268**: 16887–16890.
8. Yang BC, Mehta JL. Platelets increase the tone of quiescent rat aortic rings by release of serotonin and potentiate the subsequent contractile response to norepinephrine. *J Cardiovasc Pharmacol* 1994; **23**: 387–394.
9. Rees DD, Palmer RMJ, Schultz R, Hodson HF, Moncada S. Characterization of three inhibitors of endothelial nitric oxide synthase in vitro and in vivo. *Br J Pharmacol* 1990; **101**: 746–752.
10. Selye H, Somogyi A. Effect of cyproheptadine upon acute inflammation produced by various agents. *Med Pharm Exp* 1967; **17**: 255–263.
11. Horsfield GI. The effect of compound 48/80 on the rat mast cell. *J Path Bact* 1965; **90**: 599–605.
12. Bruns RF, Fergus JH, Badger EW, Bristol JA, Santay LA, Hartman JD, Hays SJ, Huang CC. Binding of the $A_1$-selective adenosine antagonist 8-cyclopentyl-1,3-dipropylxanthine to rat brain membranes. *Naunyn-Schmiedeberg's Arch Pharmacol* 1987; **335**: 59–63.
13. Daly JW. Adenosine receptors: targets for future drugs. *J Med Chem* 1982; **25**: 197–207.
14. Hamilton HW, Taylor MD, Steffen RP, Haleen SJ, Bruns RF. Correlation of adenosine receptor affinities and cardiovascular activity. *Life Sci* 1987; **41**: 2295–2302.
15. Gallo-Rodriguez C, Ji X-D, Melman N, Siegman BD, Sanders LH, Orlina J, Fisher B, Pu Q, Olah ME, van Galen PJM, Stiles GL, Jacobson KA. Structure-activity relationships of $N^6$-benzyladenosine-5′-uronamides as $A_3$-selective adenosine agonists. *J Med Chem* 1994; **37**: 636–646.
16. Moncada S, Palmer RMJ, Higgs EA. Nitric oxide: physiology, pathophysiology, and pharmacology. *Pharmacol Rev* 1991; **43**: 109–142.
17. Dart C, Standen NB. Adenosine-activated potassium current in smooth muscle cells isolated from the pig coronary artery. *J Physiol* 1993; **471**: 767–786.
18. Kurachi Y. G protein regulation of cardiac muscarinic potassium channel. *Am J Physiol* 1995; **269**: C821–C830.
19. Collis, MG, Hourani SMO. Adenosine receptor types. *Trends Pharmacol Sci* 1993; **14**: 560–566.
20. Jacob MI, Berne RM. Metabolism of purine derivatives by the isolated cat heart. *Am J Physiol* 1960; **198**: 322–326.
21. Rubio R, Berne RM, Katori M. Release of adenosine in reactive hyperemia of the dog heart. *Am J Physiol* 1969; **216**: 56–62.
22. Dobson JG Jr, Rubio R, Berne RM. Role of adenine nucleotides, adenosine and inorganic phosphate in the regulation of skeletal muscle blood flow. *Circ Res* 1971; **29**: 375–384.
23. Berne RM, Rubio R, Curnish RR. Release of adenosine from ischemic brain. Effect on cerebral vascular resistance and incorporation into cerebral adenine nucleotides. *Circ Res* 1974; **35**: 262–271.
24. Schrader J, Haddy FJ, Gerlach E. Release of adenosine, inosine and hypoxanthine from the isolated guinea pig heart during hypoxia, flow-autoregulation and reactive hyperemia. *Pflügers Arch* 1977; **369**: 1–6.
25. Jones CE, Mayer LR. Nonmetabolically coupled coronary vasodilatation during inosine infusion in dogs. *Am J Physiol* 1980; **238**: H569–H574.
26. Sakai K, Akima M, Matsushita H. Femoral vascular responses to purine and pyrimidine derivatives: release of 5-hydroxytryptamine by purine derivatives in isolated, cross-circulated rat hindlimb. *Japan J Pharmacol* 1979; **29**: 243–251.
27. Burnstock G, Lincoln J, Feher E, Hopwood AM, Kirkpatrick K, Milner P, Ralevic V. Serotonin is localized in endothelial cells of coronary arteries and released during hypoxia: a possible new mechanism for hypoxia-induced vasodilatation of the rat heart. *Experientia* 1988; **44**: 705–707.
28. Marquardt DL, Parker CW, Sullivan TJ. Potentiation of mast cell mediator release by adenosine. *J Immunol* 1978; **120**: 871–878.
29. Hughes PJ, Holgate ST, Church MK. Adenosine inhibits and potentiates IgE-dependent histamine release from human lung mast cells by an $A_2$-purinoceptor mediated mechanism. *Biochem Pharmacol* 1984; **33**: 3847–3852.
30. Church MK, Hughes PJ, Vardey CJ. Studies on the receptor mediating cyclic AMP-independent enhancement by adenosine of IgE-dependent mediator release from rat mast cells. *Br J Pharmacol* 1986; **87**: 233–242.
31. Lohse MJ, Maurer K, Gensheimer H-P, Schwabe U. Dual actions of adenosine on rat peritoneal mast cells. *Naunyn-Schmiedeberg's Arch Pharmacol* 1987; **335**: 555–560.
32. Northover AM, Northover BJ. Degranulation of rat omental mast cells by $A_1$ receptor agonists *in vitro*. *Mediators Inflamm* 1996; **5**: 341–345.
33. Raud J, Dahlén S-E, Smedegård G, Hedqvist P. An intravital microscopic model for mast cell-dependent inflammation in the hamster cheek pouch. *Acta Physiol Scand* 1989; **135**: 95–105.
34. Fozard JR, Pfannkuche H-J, Schuurman H-J. Mast cell degranulation following adenosine $A_3$ receptor activation in rats. *Eur J Pharmacol* 1996; **298**: 293–297.
35. Sanyal RK, West GB. The role of histamine and 5-hydroxytryptamine in injury. *Int Arch Allergy* 1965; **26**: 362–372.
36. Glover WE, Marshall RJ, Whelan RF. The antagonism of the vascular effects of 5-hydroxytryptamine by BOL 148 and sodium salicylate in the human subject. *Br J Pharmacol* 1957; **12**: 498–503.
37. Prentice DJ, Hourani SMO. Activation of multiple sites by adenosine analogues in the rat isolated aorta. *Br J Pharmacol* 1996; **118**: 1509–1517.
38. Patel M, Ramage AG. Investigation of the haemodynamic effects of the selective adenosine $A_3$ receptor agonist iodobenzyl-5-N-methyl carboxamidoadenosine, IB-MECA, in anaesthetized cats. *Br J Pharmacol* 1997; **120**: 16P.

**Received 6 January 1997; accepted in revised form 3 February 1997**
